# *Trichoderma asperelloides* Spores Downregulate *dectin1/2* and *TLR2* Receptors of Mice Macrophages and Decrease *Candida parapsilosis* Phagocytosis Independent of the M1/M2 Polarization

**DOI:** 10.3389/fmicb.2017.01681

**Published:** 2017-09-07

**Authors:** Andréa G. dos Santos, Érica A. Mendes, Rafael P. de Oliveira, Ana M. C. Faria, Aurizangela O. de Sousa, Carlos P. Pirovani, Fernanda F. de Araújo, Andréa T. de Carvalho, Marliete Carvalho Costa, Daniel Assis Santos, Quimi V. Montoya, Andre Rodrigues, Jane L. dos Santos

**Affiliations:** ^1^Programa de Pós-Graduação em Biologia e Biotecnologia de Microrganismos, Universidade Estadual de Santa Cruz Ilhéus, Brazil; ^2^Departamento de Microbiologia, Instituto de Ciências Biológicas, Universidade de São Paulo São Paulo, Brazil; ^3^Instituto Federal do Paraná Palmas, Brazil; ^4^Departamento de Microbiologia, Instituto de Ciências Biológicas, Universidade Federal de Belo Horizonte Belo Horizonte, Brazil; ^5^Departamento de Ciências Biológicas, Universidade Estadual de Santa Cruz Ilhéus, Brazil; ^6^Grupo Integrado de Pesquisas em Biomarcadores, Centro de Pesquisas René Rachou, Fundação Oswaldo Cruz Belo Horizonte, Brazil; ^7^Programa de Pós-Graduação em Sanidade e Produção Animal nos Trópicos, Universidade de Uberaba Uberaba, Brazil; ^8^Departamento de Bioquímica e Microbiologia, Instituto de Biociências, Universidade Estadual de São Paulo Rio Claro, Brazil

**Keywords:** biocontrol agent, phagocytosis, conidia, PRRs, *Candida parapsilosis*

## Abstract

The intensive use of pesticides to control pests in agriculture has promoted several issues relating to environment. As chemical pesticides remain controversial, biocontrol agents originating from fungi could be an alternative. Among them, we highlight biocontrol agents derived from the fungi genus *Trichoderma*, which have been documented in limiting the growth of other phytopathogenic fungus in the roots and leaves of several plant species. An important member of this genus is *Trichoderma asperelloides*, whose biocontrol agents have been used to promote plant growth while also treating soil diseases caused by microorganisms in both greenhouses and outdoor crops. To evaluate the safety of fungal biological agents for human health, tests to detect potentially adverse effects, such as allergenicity, toxicity, infectivity and pathogenicity, are crucial. In addition, identifying possible immunomodulating properties of fungal biocontrol agents merits further investigation. Thus, the aim of this study was to evaluate the effects of *T. asperelloides* spores in the internalization of *Candida parapsilosis* yeast by mice phagocytes, in order to elucidate the cellular and molecular mechanism of this interaction, as a model to understand possible *in vivo* effects of this fungus. For this, mice were exposed to a fungal spore suspension through-intraperitoneal injection, euthanized and cells from the peripheral blood and peritoneal cavity were collected for functional, quantitative and phenotypic analysis, throughout analysis of membrane receptors gene expression, phagocytosis ability and cells immunophenotyping M1 (CCR7 and CD86) and M2 (CCR2 and CD206). Our analyses showed that phagocytes exposed to fungal spores had reduced phagocytic capacity, as well as a decrease in the quantity of neutrophils and monocytes in the peripheral blood and peritoneal cavity. Moreover, macrophages exposed to *T. asperelloides* spores did not display the phenotypic profile M1/M2, and had reduced expression of pattern recognition receptors, such as TLR2, dectin-1 and dectin-2, all involved in the first line of defense against clinically important yeasts. Our data could infer that *T. asperelloides* spores may confer susceptibility to infection by *C. parapsilosis*.

## Introduction

The intensive use of pesticides to control biological plagues has promoted several human health problems. Indeed, previous studies have demonstrated an association between pesticide exposures and human respiratory diseases (Sanborn et al., 2002; Sankoh et al., 2016; Ye et al., 2017), including asthma (Hoppin et al., 2009; Henneberger et al., 2014) and lung cancer (Alavanja et al., 2004; Bonner et al., 2016).

The fungus genus *Trichoderma* has been used as an alternative for biological defensives, being able to limit growth of phytopatogenic fungus in roots, leaves and in soil (Ha, 2010). In addition, it has been used as a biofertilizer in several crops (Marín-Guirao et al., 2016). Hence, *Trichoderma* is an agent of choice for fungus biological control, representing 60% of the biofungicides used in agriculture nowadays (Mukherjee et al., 2012).

The *Trichoderma asperelloides*, for example, is a biological agent used as biofertilizer that promotes plant growth, being also used to treat soil diseases caused by microorganisms as *Pythium, Rhizoctonia, Fusarium, Sclerotina*, and *Phytophthora* in both green houses and crops. It is already commercialized as TRICHO PLUS Biofungicide^®^ in Africa, aiming to better develop the agricultural crops of these countries (US Environmental Protection Agency [US EPA], 2015). Generally, biocontrol agents are considered more suitable and secure than conventional pesticides. However, the inherent properties of products that come from biological systems can affect ecological cycles and organisms not targeted for the control, including the human population (Castro et al., 2001; Baxi et al., 2016).

Previous studies have already shown adverse effects in non-target populations caused by natural microorganisms used as biological control (Castro et al., 2001). Although there are no reports about human diseases associated with *T. asperelloides*, some studies demonstrated an association with other species from *Trichoderma* genus (Druzhinina et al., 2008; Kantarcioğlu et al., 2009; Santillan et al., 2011). It is well known that *Trichoderma* infection leads to a poor prognosis with high mortality ratio in a disseminated infection. Alanio et al. (2008), for example, described an opportunistic invasive lung infection in individuals with acute lymphoblastic leukemia caused by the fungus *Trichoderma longibrachiatum*. An additional study showed that a 64 year-old female patient, who was admitted in the intensive care unit with abdominal sepsis, had positive results for *Trichoderma* and *Aspergillus* in her bronchoalveolar lavage (Ariese et al., 2013).

The immune mechanisms triggered by biocontrol agents and the components of the innate and adaptive immune responses associated with *Trichoderma* infection are still unknown. Despite the induction of discrete pulmonary infiltrates, the intra-nasal administration of *T. stromaticum* spores in mice inhibited the local production of interleukin-10 (IL-10) and IFN-gamma (IFN-γ) in spleen cells (Alves-Filho et al., 2011). In addition, these authors showed *in vitro* that interactions with these fungus spores were capable to negatively regulate fungal phagocytes.

Professional phagocytes including neutrophils, monocytes and macrophages and their receptors Toll-like Receptor 2 (TLR2), Toll-like Receptor 4 (TLR4), dectin 1 and dectin 2, as well as mannose and glycan motifs, mediate mammalian innate antifungal defense against pathogen-associated molecular patterns (PAMP) into fungal cellular walls (Romani, 2004). Typically, after the phagocyte receptors bind to the PAMP, the fungus is internalized and eliminated within phagolysosomes through the action of enzymes and microbicide molecules as reactive oxygen species (Jouault et al., 2009; Dementhon et al., 2012).

Maintaining a effective immune response is essential for an organism’s proper control of a pathogenic infection. In this context, induction of M1 inflammatory macrophages have been shown to be protective against several fungal species, including *Cryptococcus neoformans*, *Aspergillus fumigatus*, and *Candida albicans* (Wagener et al., 2017). The antimicrobial properties of M1 macrophages are also linked to the up-regulation of biochemical factors, microbicidal factors producing enzymes, including the inducible nitric oxide synthase (iNOS). More, these cells have high ability to secrete interleukin 1 beta (IL-1β), tumor necrosis factor (TNF) and interleukin 12 (IL-12); and express high levels of major histocompatibility complex II (MHC-II), the CD68 marker, and co-stimulatory molecules such as CD80 and CD86 (Wager and Wormley, 2014; Cháves-Galán et al., 2015).

Alternatively, activated macrophages have the augmented ability to suppress *C. albicans* hypha formation, but they fail to eliminate the fungus from the host. Similar to *C. neoformans*’ ability to propagate the fungus in the infected organism, the M2 macrophage might contribute to cryptococcal dissemination (Wager and Wormley, 2014; Khanna et al., 2016; Wagener et al., 2017). One explanation is that the M2 cells do not upregulate iNOS but instead upregulate arginase (Arg-1), which utilizes L-arginine through a non-fungicidal pathway (Voelz et al., 2009; Khanna et al., 2016). Other genes induced by M2 cells include the Chitinase-Like Protein (Ym) that is produced in murine lungs during Th2-type immune responses and unremarkably observed as crystals at the site of microorganism infections (Wager and Wormley, 2014; Cháves-Galán et al., 2015). In addition, variable immunomodulatory mechanisms associating IL-10 to other immune system functions were shown for different fungus species. Thus, the macrophages polarization status is a key factor in fungal defense (Voelz et al., 2009; Khanna et al., 2016).

Moreover, antifungal immunity relies on many different immune pathways. Functional defects in immune pathways have been associated with the occurrence or increased severity of invasive fungal infections (Khanna et al., 2016). There are few reports on changes in immune pathways induced by pathogenic or non-pathogenic species of fungus from *Trichoderma* genus. However, due to the spores of species such as *T. viride*, *T. harzianum*, *T. stromaticum* and *T. asperelloides* (US Environmental Protection Agency [US EPA], 2015; Konstantinovas et al., 2017) being used in agriculture close to human settlements, more studies need to be done to improve our understanding of its effects on mammalian health, allowing security application and protocols improvement. Thus, in the present work we have described the effect of *T. asperelloides* on innate immune cells of C57BL/6 mice.

## Materials and Methods

### Mice and Reagents

Male C57BL/6 mice, 7–8 weeks old and weighing 25 g were purchased from the Bioterism Center of Federal University of Minas Gerais, Belo Horizonte, MG, Brazil, and maintained under standard conditions in the animal facility of the university. Experimental protocols were conducted according to institutional guidelines for animal ethics and were approved by the Animal Ethics Committee under the protocol numbers 019*/*2011 and 035/2017 State University of Santa Cruz, Ilhéus, Ba, Brazil. Fetal bovine serum (FBS) was obtained from Cultilab (Campinas, Brazil). Sodium thioglycollate medium was purchased from Difco (Kansas City, MO, United States). All other reagents and culture media RPMI were obtained from Sigma (St. Louis, MO, United States).

### *T. asperelloides* Spores

The *T. asperelloides* fungus (Supplementary Figures S1, S2) was cultivated in PDA medium at room temperature for 7–10 days. The spores were collected using 5 ml of phosphate buffered saline (PBS) and washed three times at 800 g. The cells were adjusted at hemocytometer chamber to 1 × 10^5^ spores in PBS or Thioglycollate.

### Experimental Design, Phagocyte Isolation and Culture

Twelve C57BL/6 mice were divided in four experimental groups with three mice in each group. received an intraperitoneal injection of PBS, PBS plus 1 × 10^5^ spores, thioglycollate medium (3%) or thioglycollate medium (3%) plus 1 × 10^5^ spores. Seventy-two hours post-infection mice were euthanized by cervical dislocation. Murine resident peritoneal cells were harvested by peritoneal wash with PBS, counted in a blood cell chamber and analyzed by flow cytometry, or used for cell culture. Experiments were conducted independently three times to confirm obtained data. See Supplementary Material (Presentation 1).

### Differential Count of White Blood Cells

Whole blood of each mouse was used for the quantitative determination of white blood cells. Differential leukocyte counts consisted of an evaluation of the total quantity for each type of leukocyte. The counting of total number of leukocytes (mm^3^) was performed on automatic blood cell analyzer ABC Vet (HORIBA^®^, United Kingdom). For mice, one blood drop was obtained by cardiac puncture and was immediately transferred to a microscope slide for a blood smear. Slides were stained using the Wright stain method (Lillie, 1972). Cell count was made using an optical microscope with a 100x magnification. For each slide, 100 cells were counted based in morphological criteria.

### Phagocyte Capacity

1 × 10^5^ cells obtained by peritoneal wash from control groups (PBS and Thioglycollate) and *T. asperelloides* spores treated groups (PBS + spores and Thioglycollate + spores) were added to a 24-well plate and incubated overnight in RPMI 1640 medium supplemented with 10% Fetal Bovine Serum (FBS), and penicillin/streptomycin (100 μg/ml) at 37°C and 5% CO_2_ saturation for cell adherence (Supplementary Figures S3, S5). After the incubation period, cells were washed once with PBS and 5 × 10^5^ *C. parapsilosis* yeasts (ATCC 22019) were added in each well for 2 h. After this period, wells were washed twice with PBS and cells on the slides were fixed with methanol and stained with May-Grünwald, followed by Giemsa. Phagocytosis was evaluated by counting the yeasts presence in each macrophage, using the software ImageJ^®^. The number of phagocytic yeast per macrophage was obtained by the correlation of yeasts present in 100 macrophages divided by the percentage of macrophages with at least one phagocytic yeast (Ghoneum and Gollapudi, 2004).

### Phenotypical Profile of Peritoneal Cells

Murine peritoneal macrophages were adjusted to 1 × 10^6^ cells/mL in buffer containing 2% of Bovine Serum Albumin (BSA). After, we pre-incubated cells during 10 min at 4°C with Fc block antibodies before staining (Purified Rat Anti-Mouse CD16/CD32 - Mouse BD Fc Block^TM^). Then, cells were incubated for 30 min with 10 μL monoclonal antibodies reactive to F4/80 APC; MHC II FITC and CD86 PE; LY6G FITC, CD206 FITC and CCR2 FITC; LY6C PERCP-CY5 and CCR7 PERCP-CY5.5 (eBioscience, United States). Phenotypic analyses were performed by flow cytometry using a Becton Dickinson FACScalibur flow cytometer (Becton Dickinson, Mountain View, CA, United States), collecting data on 10^5^ cells (gate by forward and side scatter properties) and analyzed using Flow Jo (Tree Star) software.

### RNA Extraction and cDNA Synthesis

For gene expression analysis, 2 × 10^6^ cells obtained by peritoneal washes from the control and *T. asperelloides* treated groups were seeded in 24 well plates overnight for cell adherence. After 24 h of incubation at 37°C and 5% of CO_2_, cells were washed twice with PBS and the plate with remained cells was kept at -80 until RNA extraction. RNA was extracted using *miRNeasy Mini Kit (QIAGEN)* according to manufacturer instructions. The RNA quality was evaluated by agarose gel and cDNA synthesis was made with the *kit-Strand Synthesis Supermix (INVITROGEN*).

### Gene Expression Analysis

Abundance of mRNA transcripts was analyzed by Real time PCR using an ABI Prism 7500 and the program *Sequence Detection System*^®^ (*Applied Biosystem*, United States). The gene 18S was amplified with the target gene, as an endogenous control for assay normalization. Reactions were performed in five replicates with a total reaction volume of 20 μL, with 8 μL of cDNA at a concentration of 50 ng, 2 μL of primer forward and reverse (100 mM), 10 μL of SyberGreen/ROX qPCR Master Mix 2X (*Fermentas*, United States). The amplification conditions were 50°C for 2 min, 95 for 10 min, 94 for 15 s, 60°C for 90 min. It was used the methodology 2^-ΔΔC_t_^ to quantify the gene expression (Livak and Schmittgen, 2001). Control reactions without cDNA (NTC) were used in all experiments as an internal control for each assay. The program *Dissociation Curve 1.0* (*Applied Biosystems*, United States) was used to evaluate the quality of transcripts products. **Table 1** shows the description of the primers used for each reaction.

**Table 1:** Primers for the specific genes used in the qPCR analysis.

Gene	Primer
iNOS	Forward: 5′-CAGCTGGGCTGTACAAACCTT -3′
	Reverse: 5′-CATTGGAAGTGAAGCGTTTCG-3′
IL-12p40	Forward: 5′-GACCCTGCCCATTGAACTGGC-3′
	Reverse: 5′-CAACGTTGCATCCTAGGATCG-3′
IRF5	Forward: 5′-TAGAGGCTACCCAGGAGCAA-3′
	Reverse: 5′-GCCCACTCCAGAACACCTTA-3′
Ym	Forward: 5′-GGGCATACCTTTATCCTGAG-3′
	Reverse: 5′- CCACTGAAGTCATCCATGTC-3′
FIZZ1	Forward: 5′-TCCCAGTGAATACTGATGAGA-3′
	Reverse: 5′-CCACTCTGGATCTCCCAAGA-3′
IL-10	Forward: 5′-TGGACAACATACTGCTAACC-3′
	Reverse: 5′-GGATCATTTCCGATAAGGCT-3′
Arginase I	Forward: 5′-AAAGCTGGTCTGCTGGAAAA-3′
	Reverse: 5′-ACAGACCGTGGGTTCTTCAC-3′
TLR2	Forward: 5′- GTGGTACCTGAGAATGATGTGGG-3′
	Reverse: 5′- GTTAATTAAGTCAGGAACTGGGTG-3′
TLR4	Forward: 5′-CTGGGTGAGAAATGAGCTGG-3′
	Reverse: 5′-GATACAATTCCACCTGCTGCC-3′
Dectin1	Forward: 5′-GAACCACAAGCCCACAGAAT-3′
	Reverse: 5′-CATGGCCCTTCACTCTGATT-3′
Dectin2	Forward: 5′-GCTAGCTGCTGTGATTTCCA-3′
	Reverse: 5′-TGAAACACACCGCTCTTCTG-3′
18S	Forward: 5′-TTCGTATTGCGCCGCTAGA-3′
	Reverse: 5′-CTTTCGCTCTGGTCCGTCTT-3′

### Statistical Analysis

Analyses were performed using GraphPad Prism version 5.0 software (San Diego, United States). The following tests were performed: one-way ANOVA test, followed by Tukey post-test. In all cases, significance was considered at *p* ≤ 0.05.

## Results

### *T. asperelloides* Spores Reduces Neutrophils and Monocytes from Mice Peripheral Blood

**FIGURE 1:**
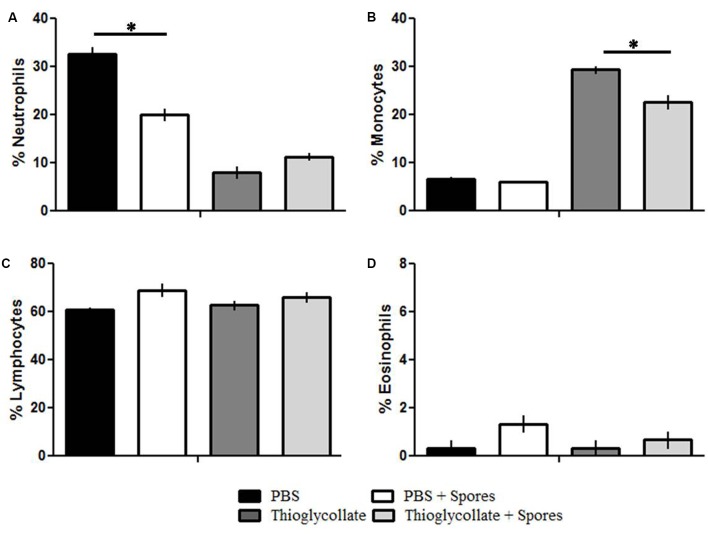
Differential count of peripheral blood leucocytes. C57BL/6 mice were treated with PBS, PBS + spore, thioglycollate and thioglycollate + spore and the differential count of peripheral blood lymphocytes were evaluated. **(A)** Neutrophils, **(B)** Monocytes, **(C)** Lymphocytes, **(D)** Eosinophil. Data represents mean ± SD of three independent experiments with three mice per group. ^∗^*p* ≤ 0.05.

In order to investigate if peripheral blood cells from C57BL/6 mice are affected by *T. asperelloides* spores, we evaluated leukocytes (neutrophils, monocytes, eosinophil, and lymphocytes) from mice blood samples submitted to different treatments. Our results showed a significant reduction of neutrophils in PBS + spores group when compared to PBS control groups (**Figure 1A** and **Table 2**). Moreover, our data showed a significant reduction of monocytes in Thioglycollate + spores group when compared to Thioglycollate control (**Figure 1B** and **Table 2**). No significant differences were observed for lymphocytes and eosinophil populations among the studied groups (**Figures 1C,D** and **Table 2**).

**Table 2:** Total number of leukocytes (mm^3^) and different leukocyte populations (segmented neutrophils, lymphocytes, eosinophils, monocytes).

	C57 BL/6			
Leukogram	PBS	PBS + Spores	Thioglycollate	Thioglycollate + Spores
Leukocytes (10^3^/μL)	3.2 ± 0.5	2.7 ± 0.7	5.5 ± 1.0	4.2 ± 1.4
Neutrophils (10^3^/μL)	1.1 ± 0.3	0.5 ± 0.2	0.3 ± 0.6	0.4 ± 0.6
Lymphocytes (10^3^/μL)	1.9 ± 0.7	2.0 ± 0.5	1.9 ± 0.8	1.8 ± 0.7
Monocytes (10^3^/μL)	0.2 ± 0.1	0.2 ± 0.2	3.3 ± 0.4	2.2 ± 0.4
Eosinophils (10^3^/μL)	0.0 ± 0.1	0.0 ± 0.1	0.0 ± 0.1	0.0 ± 0.1

*Data represent as mean ± SD from three independent experiments with three mice/group*.

### *T. asperelloides* Spores Reduces White Cells from Mice Peritoneal Cavity

**FIGURE 2:**
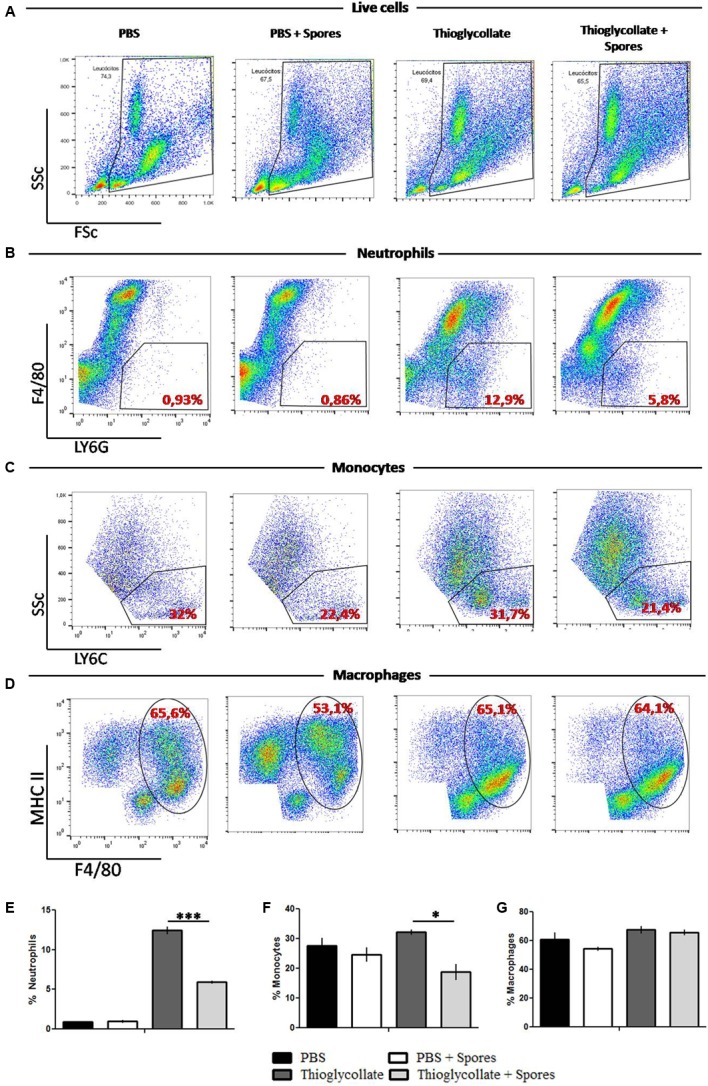
Peritoneal white cells characterization. Cells were obtained from mice peritoneal cavity treated with PBS, PBS + spores, thioglycollate or thioglycollate + spores. **(A)** Gating strategy used to identify specific cell subtypes. **(B)** Different gating strategies to identify neutrophils (LY6G^+^ F4/80^-^), **(C)** monocytes (LY6C), **(D)** macrophages (MHC II^+^ F4/80^+^). Numbers on the dot plots represent percentages of the parental cell population. **(E)** Bars from the graphic represent the frequency of neutrophils, **(F)** monocytes and **(G)** macrophages from white blood cells from mice peritoneal cavity. Data show one histogram representative of three independent experiments with three mice in each group. Results are represented as mean ± SD. ^∗^*p* ≤ 0.05, ^∗∗∗^*p* ≤ 0.0001.

The peritoneal cavity from mice was analyzed to characterize the local effect of *T. asperelloides* on immune cells. With this purpose, cells obtained from the peritoneal mice cavity 72 h after treatment were stained with monoclonal antibodies specific for diverse cell populations and quantified by flow cytometry. To select the peritoneal cell populations presented through representative density plots, specific gating strategy (Supplementary Figure S4) was performed using the forward scatter (FSc) and side scatter (SSc) (**Figure 2A**), following the analysis of neutrophils (LY6G^+^/F4/80^-^), macrophages (F4/80^+^/MHC II^+^) and monocytes (LY6C^+^) according to Misharin et al. (2012).

As shown in **Figures 2B,E**, compared with the PBS control group, the thioglycollate group presented a higher percentage of neutrophils. However, the *T. asperelloides* spores (group thioglycollate + spores) reduced two times neutrophils in the peritoneum. Regarding the monocyte population, our data showed that thioglycollate group presented a higher percentage of monocytes when compared to the thioglycollate + spores group (**Figures 2C,F**). No significant differences were observed for the macrophage population among the studied groups (**Figures 2D,G**).

### Spores of *T. asperelloides* Inhibits Phagocytosis of a Pathogenic Yeast

**FIGURE 3:**
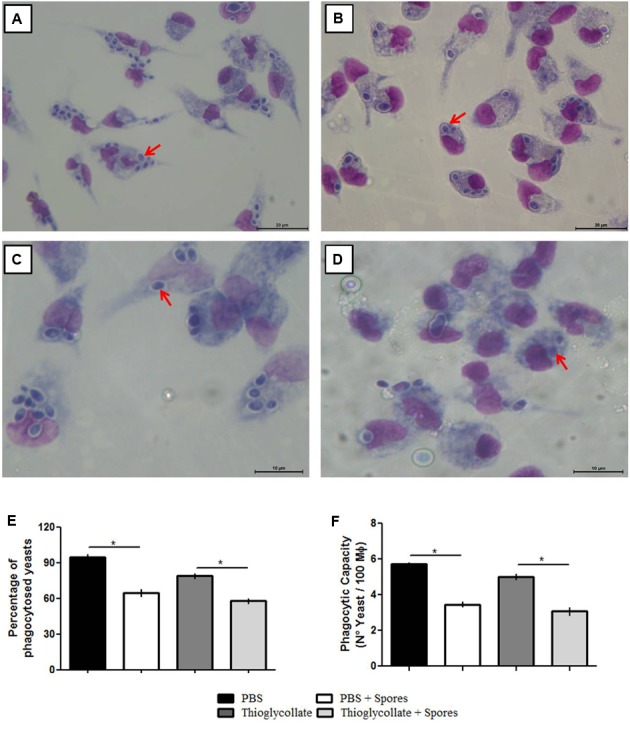
Evaluation of C57BL/6 mice peritoneal phagocytosis treated with PBS **(A)**, PBS + spores **(B)**, thioglycollate **(C)** and thioglycolatte + spores **(D)**. Graphic bars represent the percentage of *C. parapsilosis (ATCC 22019)* yeasts phagocyted by 100 macrophages **(E)** and internalized by the macrophages **(F)**. Data represent as mean ± SD from three independent experiments with three mice in each group. ^∗^*p* ≤ 0.05.

To investigate the effect of *T. asperelloides* on the functionality of the peritoneal macrophages, we conducted a phagocytosis assay with the peritoneal cells obtained from the PBS control, PBS + spore, thioglycollate, and thioglycollate + spore mice groups and challenged *in vitro* with *C. parapsilosis* yeast. The peritoneal macrophages from all groups were able to phagocytize the pathogenic yeast (**Figures 3A–D**). However, macrophages exposed to *T. asperelloides* spores presented a phagocytic inhibition. In the PBS group, 97% of cells phagocytosed the yeast compared to 70% in PBS + spores group. In the thioglycollate group 85% of cells phagocytosed the yeast compared to 55% in cells of thioglycollate + spores (**Figure 3E**). Additionally, results from the PBS group showed that macrophages phagocytosed around six yeasts, while macrophages from PBS + spores group, only three yeasts. The same phagocytic inhibition was observed in the group treated with spores + thioglycollate (**Figure 3F**), demonstrating that *T. asperelloides* spores negatively modulated the function of mice peritoneal macrophages.

### Characterization of M1/M2 Mice Peritoneal Macrophage Genetic Expression Profile When Exposed to *T. asperelloides* Spores

**FIGURE 4:**
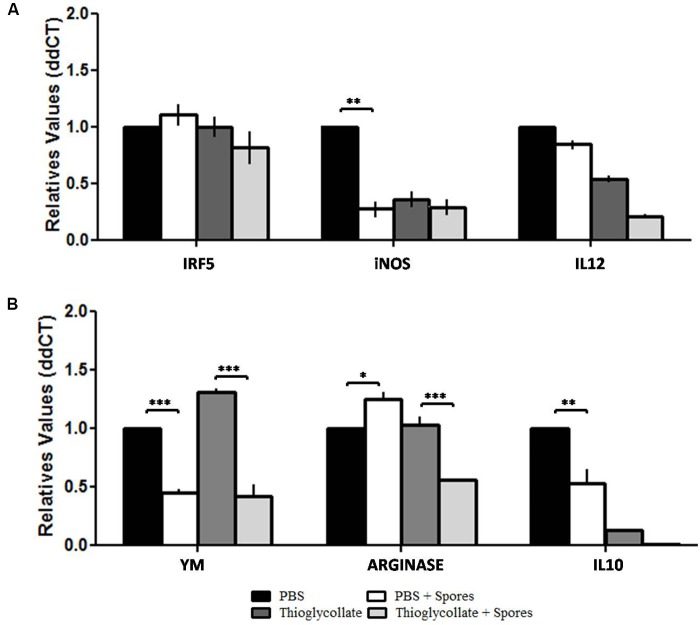
Transcriptional levels of **(A)** IRF5, iNOS and IL12, **(B)** YM, ARGINASE and IL10. M1/M2 gene expression profile of C57BL/6 peritoneal macrophages after different mice treatments (PBS, PBS + spore, thioglycollate and thioglycollate + spore) obtained by quantitative PCR. Data represented as mean ± SD. ^∗^*p* ≤ 0.05, ^∗∗^*p* ≤ 0.005, and ^∗∗∗^*p* ≤ 0.0005 from three independent experiments with three mice in each group.

Since gene expression profiles can influence a macrophage’s functionality, we investigated M1/M2 macrophage profiles and their impact on phagocytosis. For the M1 profile we analyzed the Transcription Factor Interferon Regulatory Factor 5 (*IRF5*), *iNOS* and *IL12* genes. For the M2 profile, we evaluated arginase (*Arg1*), *Ym* and *IL10* genes. Our data showed no significant differences in IRF5 and IL-12 gene expression in all studied groups. However, our results demonstrated a decrease in iNOS gene expression in the PBS + spores, thioglycollate and thioglycollate + spores groups when compared to the PBS control group, with a significant difference between the PBS and PBS + spores groups (**Figure 4A**). Moreover, in mice of PBS + spores-group and thioglycollate + spores-group the macrophage showed a diminished expression of Ym1 gene compared to the PBS and thioglycollate-groups. On the other hand, we observed an increase in gene expression in arginase in the PBS + spores group when compared to the PBS control and a reduction in the thioglycollate + spores group when compared to the thioglycollate control (**Figure 4B**). Furthermore, we found a decrease in IL-10 gene expression from PBS + spores, thioglycollate and thioglycollate + spores groups when we compared to the PBS control group, with significant difference between PBS and PBS + spores groups (**Figure 4B**).

**FIGURE 5:**
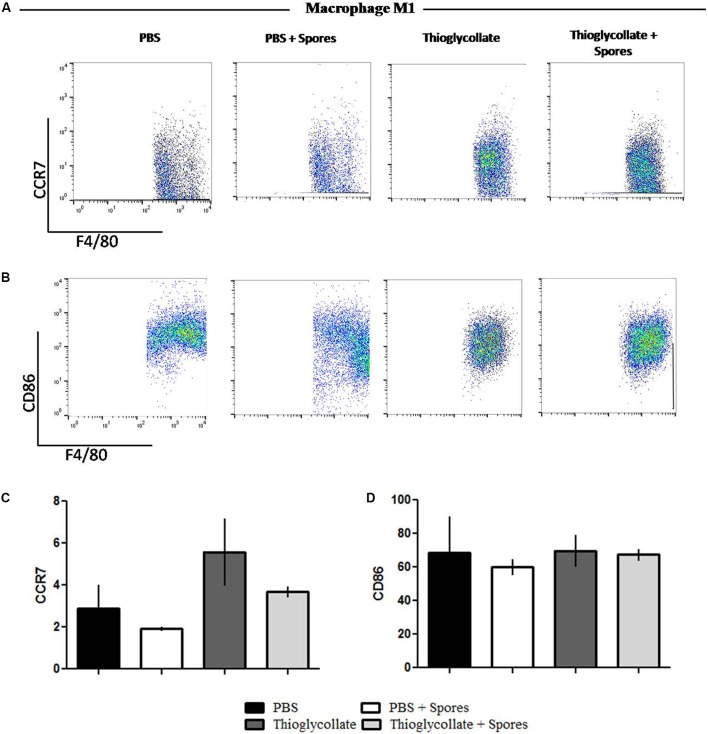
Phenotypical profile of M1 cells. Cells obtained from peritoneal wash of C57BL/6 mice treated or not with spores were collected and stained with monoclonal antibodies for CCR7 **(A)** and CD86 **(B)**. Bars from the graphic represent the median fluorescence intensity (MFI) CCR7 **(C)** and CD86 **(D)** stained cells obtained by flow cytometry. Data show one histogram representative of three independent experiments with three mice in each group. Results are represented as mean ± SD from one-way ANOVA test followed by Tukey post-test. ^∗^*p* ≤ 0.05.

**FIGURE 6:**
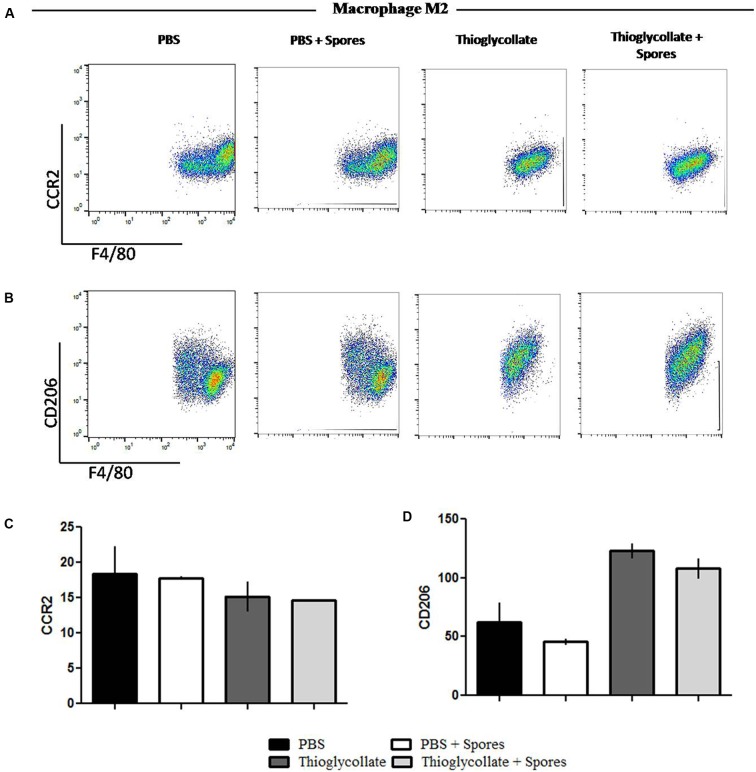
Phenotypical profile of M2 cells from C57BL6 mice peritoneal macrophages. Peritoneal cells obtained from peritoneal wash of C57BL/6 mice treated or not with spores were collected and stained with monoclonal antibodies for M1 profile, **(A)** CCR2 and **(B)** CD206. The bars from the graphics represent the median fluorescence intensity (MFI) obtained for the molecules **(C)** CCR2 e **(D)** CD206. Data show one histogram representative of three independent experiments with three mice in each group. Results are represented as mean ± SD from one-way ANOVA test followed by Tukey post-test. ^∗^*p* ≤ 0.05.

To further evaluate the effect of *T. asperelloides* spores in the profile of macrophages, cells from mice peritoneum were stained with specific surface markers (CCR2, CCR7, CD86, and CD206) were used to differentiate the MI/M2 macrophage populations. Our data showed that the chemokine receptor CCR7, typical from the M1 macrophage profile, although presented a reduction, it is not significantly altered in the groups treated with spores when comparing to their respective control group (**Figures 5A,C**). Also, no significant difference was observed in the percentage of CD86 in the presence of spores (**Figures 5B,D**). Altogether, the results suggest that *T. asperelloides* spores do not influence the M1 macrophage profile. Regarding M2 macrophage profile markers, no significant difference was observed in the percentage of CCR2 and CD206 in the presence of spores, suggesting that the spores may be unable to influence the profile of macrophages (**Figure 6**).

### *T. asperelloides* Spores Reduce PPRs in Mice Peritoneal Macrophages

**FIGURE 7:**
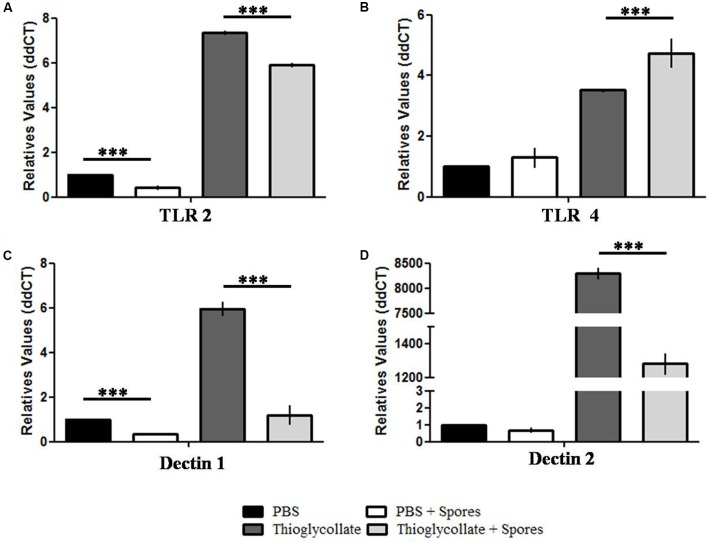
Surface receptors gene expression profile from C57BL/6 mice peritoneal macrophages. Analysis of relative expression of **(A)** TLR2, **(B)** TLR4, **(C)** dectin-1 and **(D)** dectin-2 obtained from C57BL/6 mice peritoneal macrophages exposed or not to *T. asperelloides* spores. Values were obtained by qPCR using two biological replicates from three different experiments. The relative expression was calculated using the 2^-ΔΔC_t_^ methodology (Livak and Schmittgen, 2001) and values were normalized with the 18S gene. Bars represent mean + SE (*n* = 6). ^∗∗∗^*p* ≤ 0.0005 compared to control group by Tukey post-test. Results represent data obtained from three independent experiments with three mice in each group.

Finally, in order to evaluate if the presence of *T. asperelloides* spores in the mice peritoneal cavity interfere with the genetic expression of receptors responsible for microbial recognition, we evaluated the expression of two Toll like receptors, TLR2 and TLR4, and dectin 1 and 2, a C-type lectin receptors that act in the innate immune response against fungus. Our data showed that *T. asperelloides* spores reduced expression of TLR2 in both mice groups inoculated with PBS and thioglycollate, when compared to the respective controls without spores (**Figure 7A**). On the other hand, there was augmented expression of TLR4 in the group treated with thioglycollate compared to control (**Figure 7B**). Regarding dectin receptors, our results demonstrated that the PBS + spores group presented a reduction of 50% in the expression of dectin 1 when compared to PBS group. Also, it was possible to observe in the thioglycollate + spores group a reduction of 75% when compared to control (**Figure 7C**). Moreover, a significant reduction in the transcripts of dectin-2 was observed in the thioglycollate + spores group when compared to thioglycollate group (**Figure 7D**).

## Discussion

The immune system efficacy depends on the dynamic interaction between an external agent and the competence of immunological cells, since the inability to maintain homeostasis could alter the health of the organism (Iangkovan et al., 2015). Here, we showed that organisms apparently innocuous used as biocontrol agents, as *T. asperelloides*, are able to disturb the tenuous balance of the mammal immune system.

The mice exposed to *T. asperelloides* spores decreased neutrophils in the peripheral blood. Neutrophils represent the first line defense against foreign pathogens or tissue lesions, as low numbers are associated with an increased risk of infection (Donis-Maturano et al., 2015). According to Rüping et al. (2008), patients under higher risk for *Aspergillus* infection present extended neutrophil reduction. Moreover, patients with leukemia who presented with a lower quantity of neutrophils tend to develop opportunistic fungal infections, such as disseminated candidiasis (Lionakis, 2014). Moreover, infections induced by microorganisms as bacteria, protozoa and fungus are associated with secondary neutropenia caused by extrinsic factors associated to myeloid cells in the bone marrow (Boxer and Dale, 2002). Also, there are no available reports regarding neutropenia due to fungal infection, principally related to biocontrol agents, being necessary to clarify the role and mechanism of action of *T. asperelloides* spores in the neutrophil context.

Initial neutrophil recruitment to the site of infection is followed by the recruitment of monocytes and dendritic cells. It is well known that monocytes play an important role in directing immune response. Indeed, previous studies have demonstrated that monocytes are essential during the first 48 h of fungus infection for systemic candidiasis (Ngo et al., 2014), as monocyte recruitment has been associated with the detection of yeast surface molecules by cell immune receptors such as dectin and TLRs (Sigrid et al., 2008). In fact, the present work showed that the expression of toll-like receptors essential for microbial recognition, such as *dectin 1*, *dectin 2* and *TLR2* are reduced, indicating that spores from *T. asperelloides* seem to interfere in the recruitment of immune cells for the site of inoculation. Therefore, reduction in the number of monocytes induced by *T. asperelloides* in mice suggests that mammals could develop susceptibility to other microorganisms infections after being in contact with this fungicide, which may be a concern for the health of workers exposed to it.

The expression of surface membrane receptors involved in cell migration, antigen presentation, internalization of microorganism and internal signal activation are used to classify the phenotype of macrophages as M1 inflammatory and M2 as anti-inflammatory and which presents deficiency in phagocytosis (Tarique et al., 2015). The alterations in phenotypic and functional aspects of macrophages can compromise the organism’s immune defense against pathogens (Lionakis, 2014). Our results showed that macrophages from mice exposed to *T. asperelloides* spores have a reduced ability to phagocytize yeast with clinical importance, such as *C. parapsilosis*. Regarding the M1 macrophage profile, the presence of *T. asperelloides* did not change *IRF5* expression. IRF5 is an important transcriptional factor that is able to activate directly or indirectly 20 genes from M1 profile as *IL-12*, *TNF* and *iNOS*. Furthermore, IRF5 acts as an inhibitory signaling receptor for 19 genes of cytokines and chemokines from the M2 macrophage profile, including *Arg1*, *Ym1* and *Fizz1* (Inflammatory Zone One) (Krausgruber et al., 2011). Our results showed that the expression of iNOS was reduced, while arginase transcription was augmented. Typically, the protein products of both genes use L-arginine as a substrate for nitric oxide (NO) and ornithine synthesis. While NO is intrinsically related to the defense against pathogens, the ornithine helps invaders evade macrophages (Mills and Ley, 2014). Furthermore, Alves-Filho et al. (2011) demonstrated that the expression of *iNOS* and NO production were completely inhibited in BALB/c peritoneal macrophages that were activated *in vitro* with IFN-γ + LPS and exposed to *T. stromaticum* spores. The findings regarding the expression of iNOS and arginase suggests that *T. asperelloides* spores could polarize the macrophages of C57BL/6 mice to the M2 macrophage profile. However, the inhibited expression of the Ym1 and IL10 genes, as well the absence of alterations in surface receptors CCR7, CD80 (M1), CCR2 and CD206 (M2) in the presence of the spores, leads to the need of complementary studies with the capability to support further investigations.

Although *T. asperelloides* spores did not alter the phenotypic profile of peritoneal macrophages, our data did show a reduction of the phagocytic ability of cells exposed to fungus, suggesting that phagocytosis could be independent of the M1/M2 profile. Since several studies have shown the importance of PPRs, as TLR2, TLR4, dectin 1 and dectin 2, for phagocytosis (Landmann et al., 2012; Goodridge et al., 2012; Loures et al., 2015) we demonstrated that peritoneal macrophages exposed to *T. asperelloides* spores reduced gene expression of PRRs. These receptors are considered the first defense line against pathogens, allowing recognition of the phagocytosed pathogen, and the initiation of the inflammatory process (Plato et al., 2015). Some studies have also shown the essential role of TLR2 for phagocytosis regulation during yeast internalization, phagosome maturation, and inflammatory cytokine production (Jouault et al., 2009; Moretti and Blander, 2014). In addition, Netea et al. (2010) have demonstrated that knockout mice for TLR2 are more susceptible to *C. albicans* infections.

Recent studies have described that dectin-1 is essential for phagocytosis, since it recognizes carbohydrates present in the fungus cell wall and also participates in the production of ROS in macrophages, neutrophils and dendritic cells in response to fungus structural components. Moreover, dectin-1 helps in the production of cytokines, chemokines and lipid mediators, as TNF-α, IL-1β e IL-10 (Goodridge et al., 2012; Plato et al., 2015). The present work demonstrated a reduction in IL-10 gene expression that may be due to diminished dectin-1 gene expression. In addition, macrophages exposed to spores also presented a reduction in dectin-2. Ifrim et al. (2014) showed that knockout mice for dectin 2 exhibited neutrophil and macrophage deficiency during the phagocytic process and death of *C. glabrata*, as well as an increased susceptibility to *C. albicans* infections (Saijo et al., 2010).

Here, we demonstrated that the exposure of *T. asperelloides* spores in mice reduced the quantity of neutrophils and monocytes. Additionally, peritoneal macrophages showed a reduction of the phagocytic ability and also presented reduced gene expression of PRRs receptors and IL-10, suggesting that the reduced ability to phagocyte clinical yeasts may occur by the commitment of surface receptors related with fungus detection. Together, our findings suggest a failure in phagocytosis allowing opportunistic fungus to establish infections.

## Author Contributions

AGdS and JdS conceived and designed the experiments; AGdS, MC, and DAS performed the experiments; AGdS, AOdS, and CP performed experiments and analysis of gene expression; AGdS, RdO, FdA, AdC, and AF performed the experiments and flow cytometry analysis; QM and AR made the identification and analysis of the fungus; AGdS, EM, FdA, and JdS wrote the paper.

## Conflict of Interest Statement

The authors declare that the research was conducted in the absence of any commercial or financial relationships that could be construed as a potential conflict of interest. The reviewer AF and handling Editor declared their shared affiliation.
