# Supplementary material for: Trichoderma asperelloides Spores Downregulate dectin1/2 and TLR2 Receptors of Mice Macrophages and Decrease Candida parapsilosis Phagocytosis Independent of the M1/M2 Polarization
Source: Front Microbiol. 2017 Sep 7;8:1681. doi: 10.3389/fmicb.2017.01681 (PMC5594820; doi:10.3389/fmicb.2017.01681)
Supplement: Supplementary file 1 [file Presentation_1.pdf]

Supplementary Material

Manuscript title: *Trichoderma asperelloides* spores downregulate *dectin1/2* and *TLR2* receptors of mice macrophages and decrease *Candida parapsilosis* phagocitosis independent of the phenotypical M1/M2 polarization

Supplementary Methods

Identification of the *T. asperelloides* (LIBASP02) isolate

The monosporic culture of *T. asperelloides* (LIBASP02), fungal obtained from the microbial collection of the Agro-industrial and Applied Microbiology Laboratory was identified using morphological and molecular markers. The macroscopic characteristics of the colonies (growth radius, mycelium color, presence of soluble pigment, concentric rings, and pustules) were evaluated on PDA (Potato Dextrose Agar), CMA (Corn Meal Agar) and SNA (Synthetic Nutrient Agar) after seven days of incubation at 25 °C, in darkness. Also, growth rates were determined on the three culture media incubated at three different temperatures (25, 30 and 35°C).

For analysis of the microscopic characteristics (shape and measurements of conidiophores, supporting cells, phialides, conidia, and chlamydospores), slide cultures were performed in PDA and CMA and incubated at 25 °C for 72 hours. All slides were prepared using 3M KOH (Potassium hydroxide) and observed under a microscope.

For molecular analysis, genomic DNA was extracted from the culture incubated at 25 °C in PDA for three days, in darkness (MONTOYA *et al.*, 2016). The partial sequence (ca. 563–615 bp) of the gene encoding for the elongation factor 1 alpha (tef1) was amplified. Amplification conditions and primers used were described according to Atanasova et al. (2013). The obtained amplicons were purified and sequenced by the Sanger method in an ABI 3500 (Life Technologies). The obtained forward and reverse sequences were assembled in BioEdit (HALL, 1999) and the consensus sequence (SAMUELS et al., 2010) was aligned with other phylogenetically related fungal sequences obtained from NCBI-GenBank in MAFFT (KATOH & STANDLEY, 2013). *Protodrea pallida* (CBS 29978) was used as an out group. The phylogenetic tree was reconstructed using Bayesian Inference in MrBayes v.3.2.2 (RONQUIST *et al.*, 2012). The final tree includes a total of 53 partial tef1 sequences whose final alignment presented a total size of 591 bp. The nucleotide substitution model selected for the data set (HKY + I + G) was calculated in jModelTest 2 (DARRIBA *et al.*, 2012) using the Akaike information criterion with a 95% confidence interval. Two independent runs were performed, each containing three hot chains and one cold chain; each run consisted of the Markov Chain Monte Carlo sampling (MCMC) with 200,000 generations. Finally, the first 25% of the MCMC generations was discarded as burn-in. The final tree was edited using the Adobe Illustrator CS6 (Adobe Systems).

Supplementary Results

Morphological and molecular identification of the LIBASP02 isolate

In order to identify if the species used in this work was *T. asperelloides,* after morphological analysis, the isolate showed radial growth with cottony mycelium.

Colonies turned green after 72 hours on PDA medium (Supplementary figure 1A, 1B and 1C). Likewise, the isolate formed conidiophores in the aerial mycelium with green smooth ovoid conidia in ampulliform phialides (Supplementary figure 1D, 1E and 1H). Pustules and chlamydospores were formed in all three analyzed culture media (Supplementary figure 1F and 1G). No soluble pigment was observed on all culture medium. All the morphological characteristics observed are in agreement with the description of *T. asperelloides* (SAMUELS *et al.*, 2010*).*

In addition, Blast results showed that the LIBASP02 isolate had 100% of identity with the culture collection strains GJS04-217 and GJS 99-6 (*T. asperelloides*). Also, phylogenetic analysis based on the partial tef1gene revealed that the isolate LIBASP02 formed a monophyletic group (with a high posterior provability value) with *T. asperelloides* (supplementary figure 2). This analysis showed *T. asperellum* as a phylogenetic sister clade of *T. asperelloides* (supplementary figure 2). Finally, both the morphological and molecular data supported the identification of LIBASP02 isolate as *T. asperelloides*.

Supplementary Figure Legend

Figure 1. (supplementary). **Morphological characteristics of the isolate LIBASP02**. (**A** - **C**) cultures on PDA, CMA, and SNA media, respectively, after 7 days at 25°C, **(D)** Conidiophore, **(E)** Phialides arrangement, (**F** - **G**) Chlamydospores, (**H**) Conidia.

Figure 2. (supplementary). **Phylogenetic tree of the isolate LIBASP02 (in bold), based on (tef1) marker**. The tree was reconstructed using Bayesian Inference algorithm and the numbers on the branches indicate the Monte Carlo posteriores probabilities. The tree includes a total of 53 tef1 sequences. The alignment presents 53bp. Sequences of the other *Trichoderma* species were obtained from NCBI-GenBank and from (Samuels et al. (2010). The species name are followed by the strain codes. *Protodrea pallida* CBS 29978 was used as an outgroup.

Figure 3. (supplementary). **Gating strategy from white cells**. Cells were stained with antibodies and neutrophils were identified as LY6G+ F4/80-, monocytes as LY6C+, and macrophages as MHC II+ F4/80+.

Figure 4. (supplementary). **Evaluation of C57BL/6 mice peritoneal cells**. Cells were treated with PBS (A), PBS + spores (B), thioglycollate (C) and thioglycolatte + spores (D), stained and evaluated under the microscope. Cells from peritoneal lavage were cultivated according to the described methodology (section phagocyte capacity line 218-228). Both, cells incubated with or without *Candida parapsilosis* were stained by MGG and checked under the microscope. To clarify the review question, we added a figure below showing the results obtained with cells that were not incubated with *Candida* showing absence of *T. asperelloides* spores inside macrophages. We did not observe remaining spores in the peritoneal lavage. After the incubation time, cells were washed once with PBS and $5x10^5$ C. parapsilosis yeasts (ATCC 22019) were added or not in each well for two hours" (line 218-222).

Figure 5. (supplementary). **Evaluation of the phagocytic capacity of peritoneal cells of incubated C57BL / 6 mice.** Cells were treated with PBS (A), PBS + spores (B), thioglycollate (C) and thioglycolatte + spores (D). After the incubation period, cells

were washed once with PBS and $5x10^5$ *C. parapsilosis* yeasts (ATCC 22019) were added in eachwell for 24 hours. After this period, wells were washed twice with PBS and cells on the slides were fixed with methanol and stained with May-Grünwald, followed by Giemsa. We observed that the yeast make pseudo hyphas what may be difficult to correct evaluate phagocytosis. We believe that an in vivo infection will be better to follow it up.

Supplementary References

Atanasova, L., Druzhinina, I. S., Jaklitsch, W. M. (2013). "Two hundred *Trichoderma* species recognized on the basis of molecular phylogeny," in: *Trichoderma*: biology and applications, (*CAB International*), 10 – 42. doi.org/10.1079/9781780642475.0000

Darriba, D., Taboada, G. L., Doallo, R., Posada, D. (2012). jModelTest 2: more models, new heuristics and parallel computing. *Nat Methods*. 9 (8): 772. doi: 10.1038/nmeth.2109

Hall, T. A. (1999). BioEdit 5.0.9: a user-friendly biological sequence alignment editor and analysis program for Windows 95/98/NT. *Nucleic Acids Symp Ser*. 41: 95– 98.

Katoh, K., Standley, D. M. (2013). MAFFT multiple sequence alignment software version 7: improvements in performance and usability. *Mol Biol* Evol. 30: 772 – 780. doi: 10.1093/molbev/mst010

Montoya, Q. V., Meirelles, L. A., Chaverri, P., Rodrigues, A. (2016). Unraveling Trichoderma species in the attine ant environment: description of three new taxa. *Antonie Van Leeuwenhoek*. 109 (5): 633 – 51. doi: 10.1007/s10482-016-0666-9

Ronquist, F., Teslenko, M., Van der Mark, P., Ayres, D. L., Darling, A., Hohna, S., Larget, B., Liu, L., Suchard, M. A., Huelsenbeck, J. P. (2012). MrBayes 3.2: efficient Bayesian phylogenetic inference and model choice across a large model space. *Syst Biol*. 61: 539 – 542. doi: 10.1093/sysbio/sys029.

Samuels, G. J., Ismaiel, A., Bon, M.C., Respinis, S., Petrini, O. (2010). Trichoderma asperellum sensu lato consists of two cryptic species. *Mycologia*. 102 (4): 944 – 966.
