# Supplementary figures and images for: Trichoderma asperelloides Spores Downregulate dectin1/2 and TLR2 Receptors of Mice Macrophages and Decrease Candida parapsilosis Phagocytosis Independent of the M1/M2 Polarization
Source: Front Microbiol. 2017 Sep 7;8:1681. doi: 10.3389/fmicb.2017.01681 (PMC5594820; doi:10.3389/fmicb.2017.01681)

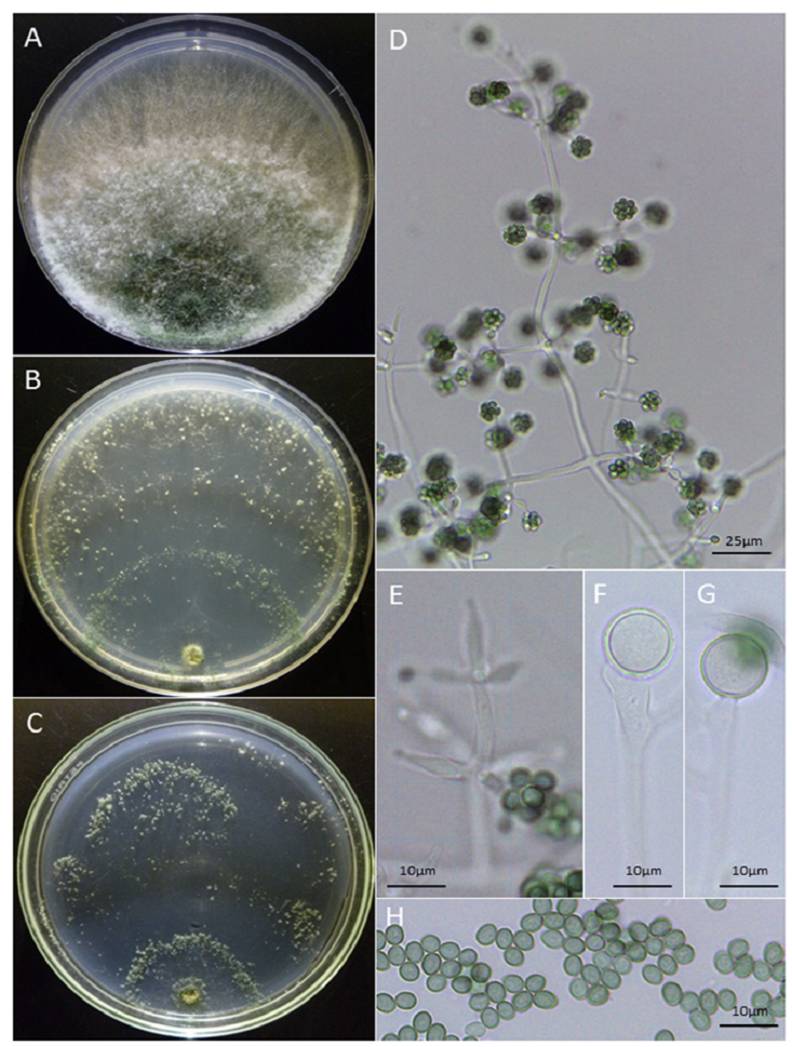

Supplement: Supplementary file 2 [file Image_1.JPEG]

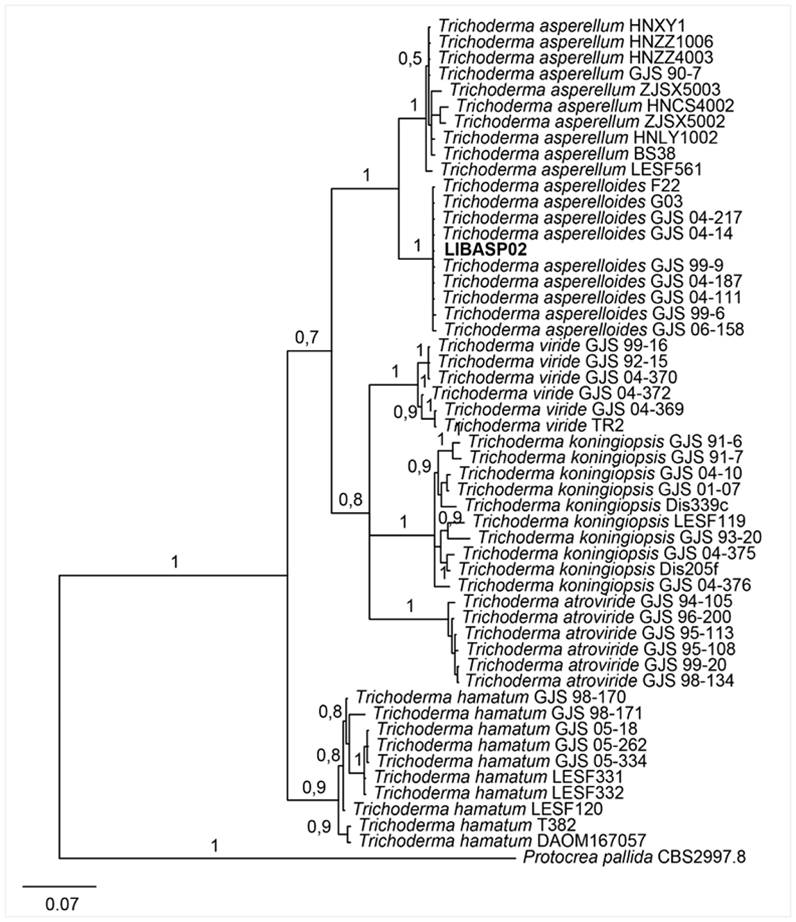

Supplement: Supplementary file 3 [file Image_2.JPEG]

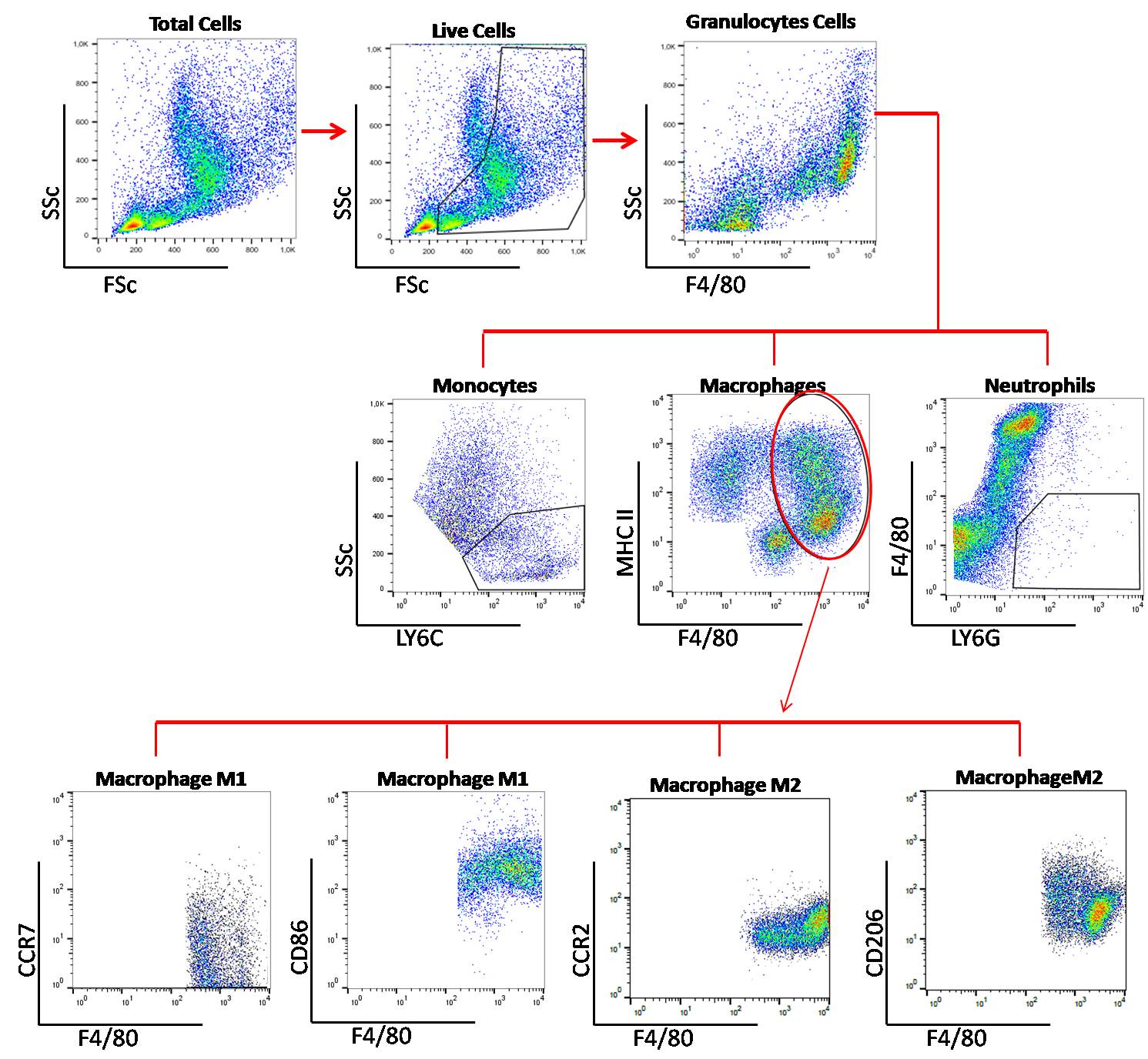

Supplement: Supplementary file 4 [file Image_3.JPEG]

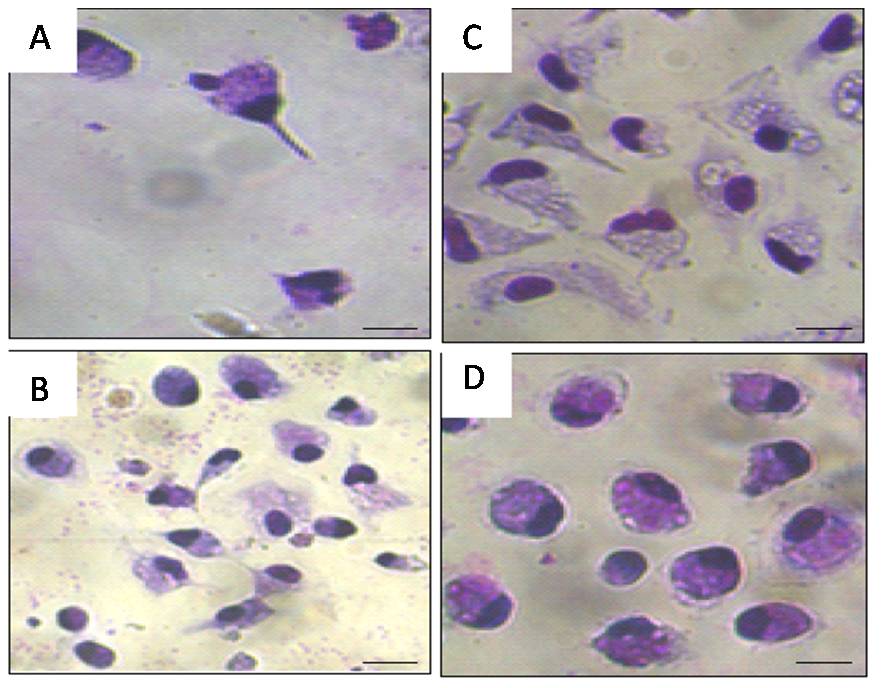

Supplement: Supplementary file 5 [file Image_4.JPEG]

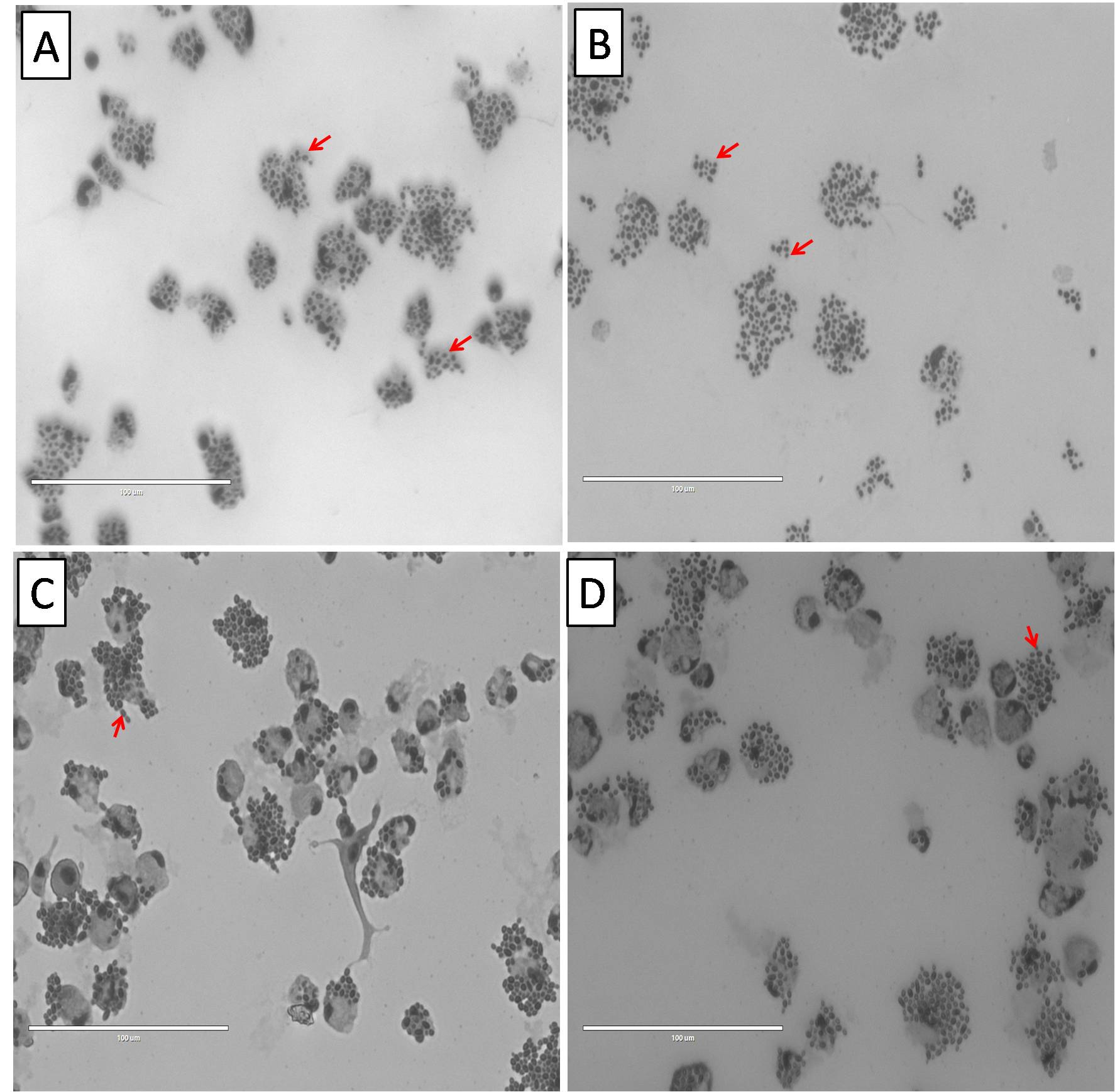

Supplement: Supplementary file 6 [file Image_5.JPEG]
